# Entrapment of organic fluorophores in calcium phosphate nanoparticles with slow release

**DOI:** 10.3906/kim-1902-57

**Published:** 2020-02-11

**Authors:** Laila SADALLAH, Aicha BOUKHRIS, Hassan HANNACHE, Said GMOUH

**Affiliations:** 1 Department of Chemistry, Faculty of Science Ben M’sik, Hassan II University of Casablanca, Casablanca Morocco; 2 Higher School of Textile and Clothing Industries, Casablanca Morocco; 3 Department of Materials Science and Nanoengineering, Mohamed VI Polytechnic University, Benguerir Morocco

**Keywords:** Calcium phosphate nanoparticles, slow release, entrapment, organic fluorophores

## Abstract

Two organic fluorophores, fluorescein (F) and rhodamine B (Rd), were entrapped in calcium phosphate nanoparticles. The as-obtained nanoparticles can be used for biological release applications. For this aim, calcium phosphate nanoparticles were synthesized using the precipitation method. Structural analysis of these nanoparticles was performed using XRD, FTIR, and Raman spectroscopy, confirming that the synthesized nanoparticles were hydroxyapatite. TEM and SEM analyses demonstrated that these nanoparticles had a size of 20 nm and a well-defined morphology. F and Rd (about 0.5 wt.%) were entrapped in these nanoparticles and their release, as a function of time, was studied via UV-Vis spectroscopy. The obtained results showed that the release of both fluorophores was progressive over time. The trapping efficiencies of the fluorophores were 67.15% and 90.76% for F and Rd, respectively.

## 1. Introduction

Calcium phosphate is one of the most important inorganic minerals in nature and it plays an essential role in our daily lives [1]. As an inorganic mineral, it represents the most indispensable constituent of human bones and teeth, and it is necessary in the function of nerves, cells, muscles, and bones. Calcium phosphate has a very wide range of applications due to its excellent biodegradability, bioresorbability, and osteoconductivity [2,3]. For instance, calcium phosphates are used as ocular implants allowing eye movement [4], nanosystems for photodynamic therapy, contrast agents for multimodal imaging [5], drug delivery systems [6], vaccine adjuvants, and antifungal agents [3]. Calcium phosphates are also present in the manufacturing of biosensors [7] and have great attraction for binding with active substances, namely proteins, antigens, vaccines, and immunogens [8,9].

Under specific conditions, including temperature, humidity, and the presence of impurities, biological calcium phosphates exist in different aspects and morphology [10]. The forms of calcium phosphate currently applicable in the biomedical field are hydroxyapatite (HAP), amorphous calcium phosphate (ACP), tricalcium phosphate (TCP), monocalcium phosphate monohydrate, biphasic calcium phosphate, and mixtures thereof. Of these forms, HAP remains the most stable, least soluble, and most versatile material for nanomedicine. It is the most appropriate biomedical material, given its unique physicochemical properties. Like other formulations, HAP can be treated in dense or porous bulk form and as powders, granules, scaffolding, or coatings [11].

For drug delivery systems and absorbable scaffolds, in most cases, the administration and monitoring of molecules alone cannot succeed. For this reason, many studies on calcium phosphate nanoparticles as carriers have been conducted [12], in which they protect active substances, prevent their degradation, help them to overcome physical barriers, and increase their selectivity. The desired properties of such nanoparticles strongly depend on their application and target tissue. The most important parameter is particle size. Particles smaller than 100 nm are hardly recognized by the immune system and can be easily taken up by cells [13].

For imaging applications, calcium phosphate nanoparticles can be used as fluorescent probes after doping with lanthanides [14–18] or surface functionalization with organic dyes [13,19–22]. Several studies have reported the use of fluorescein (F) and its derivatives. From these studies, F is considered an important fluorescent marker or probe that can be used for analyte detection and optical imaging due to characteristics such as its high emission peak intensity, high molar absorption coefficients, and quantum yields in aqueous media [23,24]. Moreover, rhodamine B (Rd) has attracted considerable attention as a marker due to its spectral properties in solution. To determine the quantum yields of fluorescence, Rd is often used as a standard [25]. It is also used as an active medium for tunable lasers [26]. Fluorescent Off/On sensors based on Rd are characterized by very high sensitivity and selectivity for the detection of changes in metal ions. It could detect them in biological systems because it is pH-independent and could recognize ions under physiological conditions. It has been successfully used for fluorescence imaging in living cells [27].

Within this context, calcium phosphate nanoparticles were synthesized herein using the precipitation method. Disodium hydrogen phosphate dehydrate and calcium chloride were used as the main precursors. The chemical structure of the obtained nanoparticles was studied via Raman, FTIR, and XRD spectroscopy. Their size and morphology were studied by TEM/SEM microscopy. Next, these as-obtained HAP nanoparticles (nano-HAP) were used to trap 2 types of fluorophores, F and Rd (about 0.5% by weight fluorophore), in order to study their release properties. The trapping efficiency was tested using UV-Vis spectroscopy. The release studies were performed as a function of time and the release efficiency for each fluorophore was calculated.

## 2. Materials and methods

### 2.1. Chemicals and materials

Disodium hydrogen phosphate dehydrate (Na2 HPO4 .2H2 O, 177.99 g/mol), calcium chloride (CaCl2 , 110.98 g/mol), sodium dodecyl sulfate (SDS) (CH3 (CH2)11 OSO3 Na, 288.38 g/mol), absolute ethanol (CH3 CH2 OH, ≥99.89%), and ultrapure water (18.02 g/mol) were all purchased commercially from Sigma-Aldrich (St. Louis, MO, USA). All reagents used in the experiments were of analytical quality.

### 2.2. Synthesis of calcium phosphate nanoparticles (nano-HAP)

The crystalline nanoparticles of the calcium phosphate (nano-HAP) were synthesized using the precipitation method. Na2 HPO4 solution (0.3 M; 60 mL) was added dropwise to 600 mL of a CaCl2 (0.05 M) solution that contained SDS (9 ×10−4 mol/L). SDS was added as a dispersant to avoid nanoparticle agglomeration during synthesis. According to the literature, to have nanoparticles with such properties in terms of size and morphology using the coprecipitation method, it is necessary to maintain the pH at a basic level of about 10.0 ±0.5 [28]. For this aim, a dilute ammonia solution (1 mol/L) was added. Next, the solution was stirred for 24 h at room temperature. The obtained suspension was first washed 3 times with ethanol and ultrapure water, then centrifuged (12,000 rpm) for 15 min and heated at 150 °C for 24 h to obtain the crystalline phase of nano-HAP and remove the ammonium ions remaining in the final product [29].

### 2.3. Entrapment of fluorophores in calcium phosphate nanoparticles

**Figure 1:**
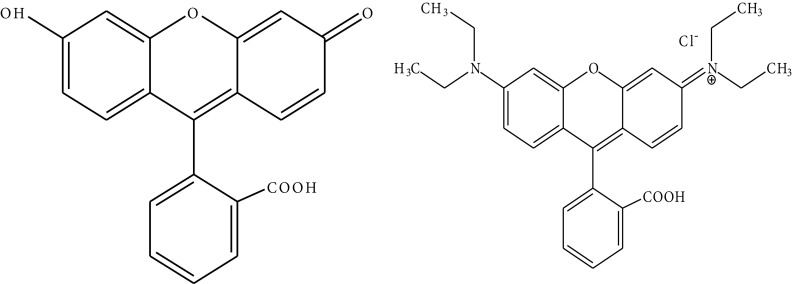
Chemical structure of F (left) and Rd (right).

During the synthesis of nano-HAP, the fluorophores were added to a CaCl2 solution (about 0.5 wt.%), resulting in the entrapment of F and Rd (Figure 1) by calcium phosphate nanoparticles, and then the suspension was heated at 150 °C for 24 h. The obtained fluorescent calcium phosphate nanoparticles were named nano-HAP-F and nano-HAP-Rd.

### 2.4. Study of fluorophore release

The study of the release of Rd from nano-HAP-Rd and F from nano-HAP-F was performed in 4 mL of water and in a mixture of water (3/4 mL) + ethanol (1/4 mL), respectively. In both studies, the solution was added to 21 mg of fluorescent calcium phosphate nanoparticles.

The study of the release was conducted within a leaching time range of 5 min to 24 h for nano-HAP-F and 5 min to 4 days for nano-HAP-Rd. The solution was filtered, placed under a UV-Vis lamp, and later analyzed via UV-Vis spectroscopy in order to determine the released concentration over time.

### 2.5. Characterization techniques

#### 2.5.1. X-ray diffraction

In order to determine the nature of synthesized nanoparticles phase, the samples were analyzed via XRD. Diffraction data were collected at room temperature on a D2 PHASER diffractometer (Billerica, MA, USA),with Bragg–Brentano geometry using CuKα radiation (λ = 1.5406 Å) at 30 kV and 10 mA. The patterns were scanned through 0.01 (2θ) steps in the 2θ range of 10°to 80°.

The sample preparation was done as follows: 400 mg of powder was placed on the specimen holder and lightly pressed with a glass slide, and then the excess powder was removed. LaB6 powder was used as the standard.

#### 2.5.2. FTIR spectroscopy

To determine the chemical structure of the nanoparticles, the sample was analyzed via FTIR spectroscopy. IR spectra were recorded between 400 and 4000 cm−1 with a Nicolet 205 FTIR spectrometer (Waltham, MA, USA).

A homogeneous mixture of approximately 1% of the sample powder/KBr was ground and deposited in a mold. It was then subjected to very high pressure in a hydraulic press to obtain powder/KBr pellet.

#### 2.5.3. Raman spectroscopy

Raman spectroscopy was performed in order to confirm the chemical structure of nano-HAP. Raman spectra were obtained on a Labram 010 spectrometer (Dilor; Horiba Scientific, Kyoto, Japan), using the 514.5-nm green line of an argon laser as an excitation source. The sample was step-scanned at 1 cm−1 resolution between 500 and 2000 cm−1 .

#### 2.5.4. Transmission electron microscopy

TEM was performed on a Hitachi H7650 microscope (Tokyo, Japan) at an accelerating voltage of 80 to 120 kV. Measurements were obtained using high-resolution mode for magnifications ranging from 4000 to 600,000.

#### 2.5.5. Scanning electron microscopy

The SEM micrographs were recorded using a Hirox SH-4000 M microscope (Tokyo, Japan) operating under high vacuum from 5 kV to 30 kV, and using a secondary electron detector (SE detector & BSE detector, multimode). The holder and samples were placed in the metallizer chamber, where a vacuum was created.

#### 2.5.6. UV-Vis spectroscopy

The entrapment efficiencies of nano-HAP-F and nano-HAP-Rd were determined by UV-Vis solid spectroscopy. A Shimadzu UV-VIS-NIR spectrophotometer (Kyoto, Japan) was used to measure the spectrum of light absorption at a wavelength ranging from 200 to 2400 nm.

The entrapment efficiency was confirmed by UV-Vis spectroscopy. A UV-1800 spectrophotometer at a resolution of 1 nm, which was designed in accordance with the European Pharmacopoeia (CFR Part 11), was used.

The release yield (Rt.%) of the 2 fluorophores was calculated by measuring the fluorophore content in the supernatant after centrifugation and filtration, and the amount entrapped by nano-HAP was deduced according to the following formula [30]:

Rt(%)=mi(Fl)-ms(Fl)mi(Fl)x100

where mi(Fl) is the initial fluorophore mass and ms(Fl) is the supernatant fluorophore mass.

## 3. Results and discussion

### 3.1. Characterization of calcium phosphate nanoparticles (nano-HAP)

**Figure 2:**
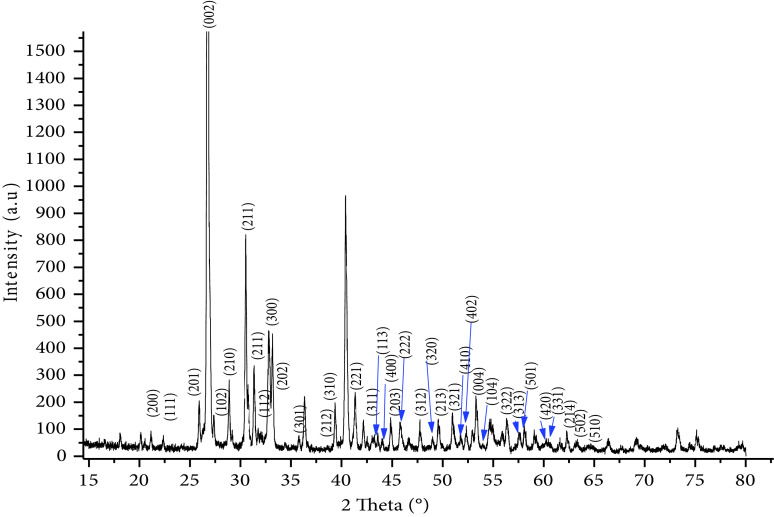
Nano-HAP XRD diffractogram.

**Figure 3:**
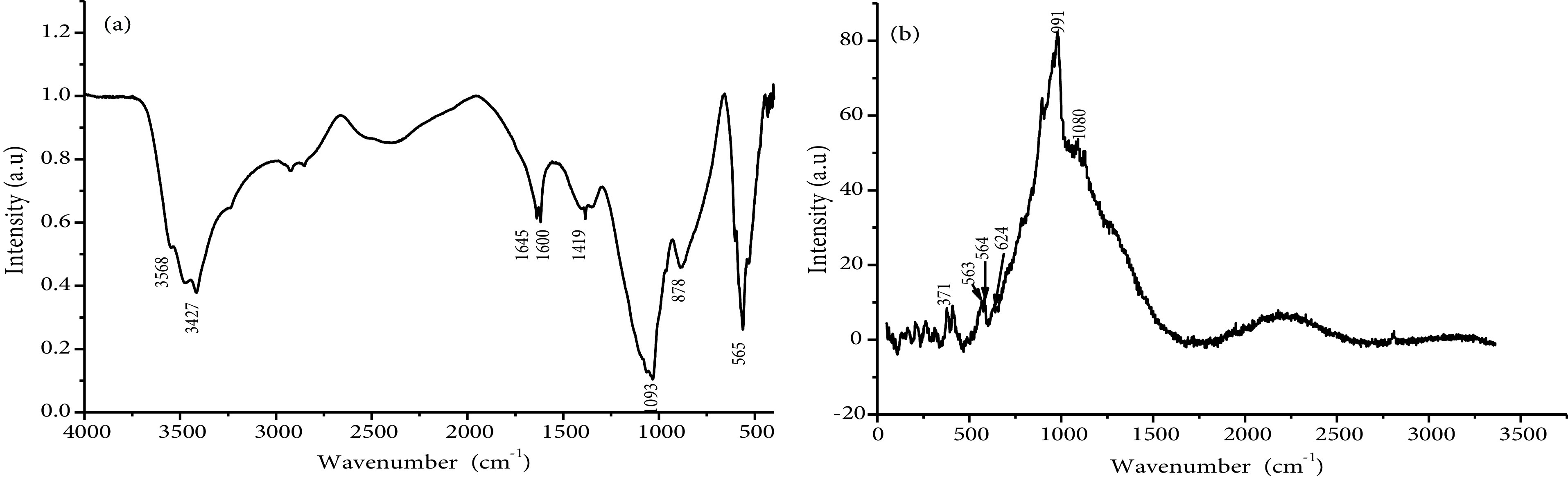
FTIR (a) and Raman (b) spectra of nano-HAP.

The synthesized calcium phosphate nanoparticles were examined by XRD, Raman, and FTIR. The results showed that the nanoparticles had a good crystalline HAP structure. According to the XRD diffractogram (Figure 2), the calcium phosphate phases, such as ACP, octacalcium phosphate, TCP, and dicalcium phosphate dehydrate, were not detected, which allowed the conclusion that the main composition of nano-HAP was the crystalline phase of the calcium phosphate (HAP). The nano-HAP had the typical XRD HAP profile and all of the diffraction peaks could be assigned according to the standard model (JCPDS09-0432). The FTIR spectrum of calcium phosphate nanoparticles (nano-HAP) (Figure 3a) showed the triple degenerate asymmetric stretching and bending vibrations of PO3−4 , which were observed only in the HAP, at 1093 and 565 cm−1 [31]. This experimental result confirmed the crystallized phase of the HAP. The CO2−3 peaks at 1419 and 871 cm−1 were due to the presence of some carbonate ions in nano-HAP. The incorporation of carbonates is a common phenomenon during the formation of biological apatites [12,32]. The peak at 3568 cm−1 represented the structural -OH group of HAPs. The peaks at 3427 and 1645 cm−1 corresponded to the remaining water. The broad peak around 1600 cm−1 could be attributed to the C-C bond of the SDS detected in the sample, which showed that there were traces of residual additives. The results were confirmed by Raman spectroscopy (Figure 3b), where the characteristic bands of the PO3−4 ion were located as follows: ν1 at 991 cm−1 , ν2 at 564 cm−1 , ν3 at 1080 cm−1 , and ν4 at 563 cm−1 [33]. Furthermore, the characteristic bands of the OH group were located at 624 cm−1 and 371 cm−1 [34,35].

**Figure 4:**
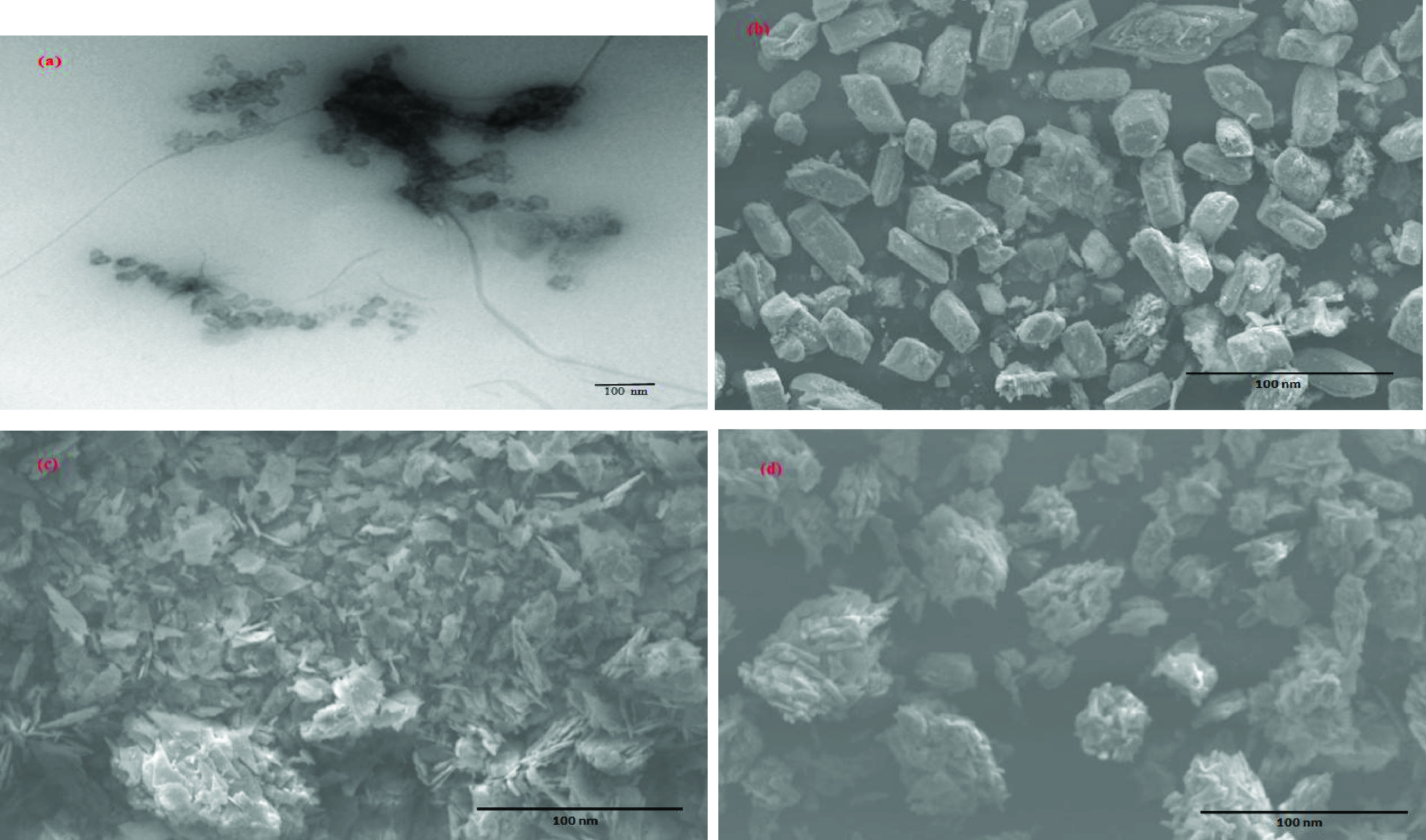
TEM image of nano-HAP (a). SEM images of nano-HAP (b), nano-HAP-F (c), and nano-HAP-Rd (d).

The morphology and size of nano-HAP were characterized by TEM and SEM. The TEM observations clearly showed that the nano-HAP particles had the morphology of grains with an approximate diameter of 20 nm (Figure 4a). The SEM observations confirmed that the particles had a uniform size, and were nanodispersed with well-defined morphology (Figure 4b).

The SEM images showed a remarkable change in morphology between the raw nanoparticles and those that trapped the fluorophores. In the case of nano-HAP (Figure 4b), nanoparticles were presented as isolated grains with well-defined nanostructured morphology. Regarding nano-HAP-F (Figure 4c) and nano-HAP-Rd (Figure 4d), it was observed that the fluorophores had been interposed between the grains of nano-HAP.

### 3.2. Characterization of nano-HAP-F and nano-HAP-Rd

**Figure 5:**
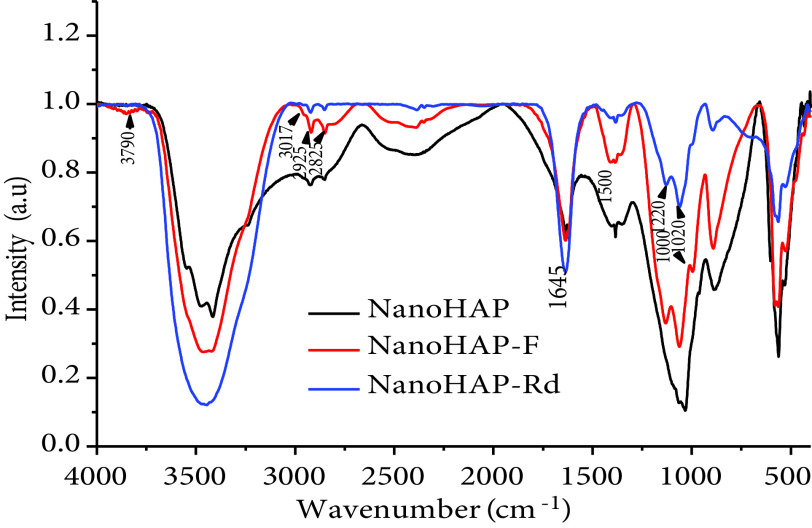
FTIR spectra of nano-HAP-F (a) and nano-HAP-Rd (b).

Characterization of the raw nanoparticles and nanoparticles entrapping F and Rd by FTIR were performed. The spectra of nano-HAP-F (Figure 5a) and nano-HAP-Rd (Figure 5b) were characterized by the appearance of an intense and wide band between 3790 and 3017 cm−1 that was associated with the hydrogen bond (interand intra-) of the OH-O group. A precise assignment of the OH stretching mode was not clear because a very intense wide band was observed in the OH region of the spectrum due to the presence of H2 O. The 2 bands that appeared at 2925 cm−1 and 2825 cm−1 for nano-HAP-F and nano-HAP-Rd can be attributed to the aliphatic CH vibration stretches of F and Rd. The bands that appeared at 1200–1000 cm−1 were attributed to the C-O stretching vibrations in alcohols and phenols, confirming the presence of the OH group of F (Figure 5a). The band at 1645 cm−1 was due to the C=C and the C=N elongation band of nano-HAP-Rd (Figure 5b). Medium intensity bands between 1020 and 1220 cm−1 were attributed to the C-N band of nano-HAP-Rd. The remarkable difference in intensity between the spectra of nano-HAP-F and nano-HAP-Rd was due to the presence of the OH and C=O groups, confirming the presence of F in nano-HAP-F.

**Figure 6:**
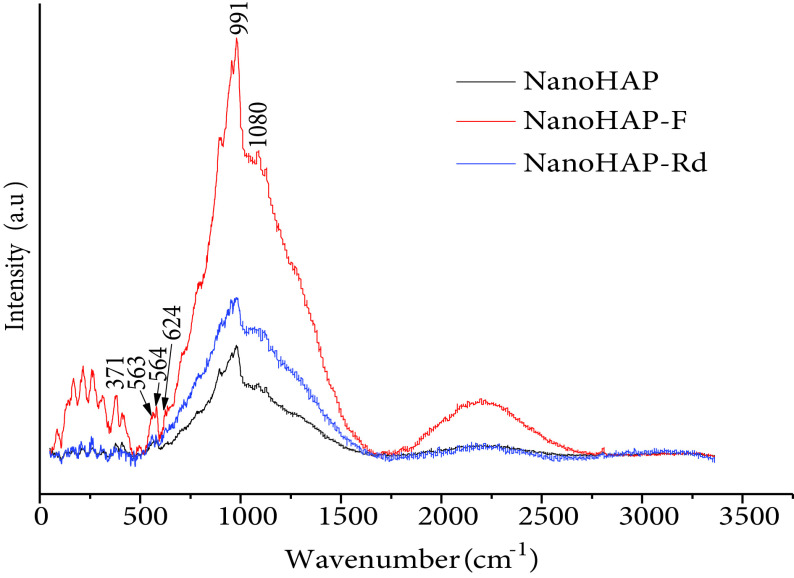
Raman spectra of nano-HAP, nano-HAP-F, and nano-HAP-Rd.

The chemical composition was confirmed by Raman spectroscopy (Figure 6), where the characteristic bands of the PO3−4 ion appeared in all 3 spectra (raw nano-HAP, nano-HAP-F, and nano-HAP-Rd) as ν1 at 991 cm−1 , ν2 at 564 cm−1 , ν3 at 1080 cm−1 , and ν4 at 563 cm−1 . The characteristic bands of the OH group were located at 624 cm−1 and 371 cm−1 . The results were in agreement with those of the FTIR spectroscopy. The difference in the peak intensity was remarkable for the results obtained by Raman spectroscopy due to the presence of the OH and C=O groups characterizing F in nano-HAP-F and the C=O group characterizing Rd in nano-HAP-Rd.

### 3.3. Fluorescence of nano-HAP-F and nano-HAP-Rd

The fluorescence of nano-HAP, nano-HAP-F, and nano-HAP-Rd were observed under a UV lamp.

**Figure 7:**
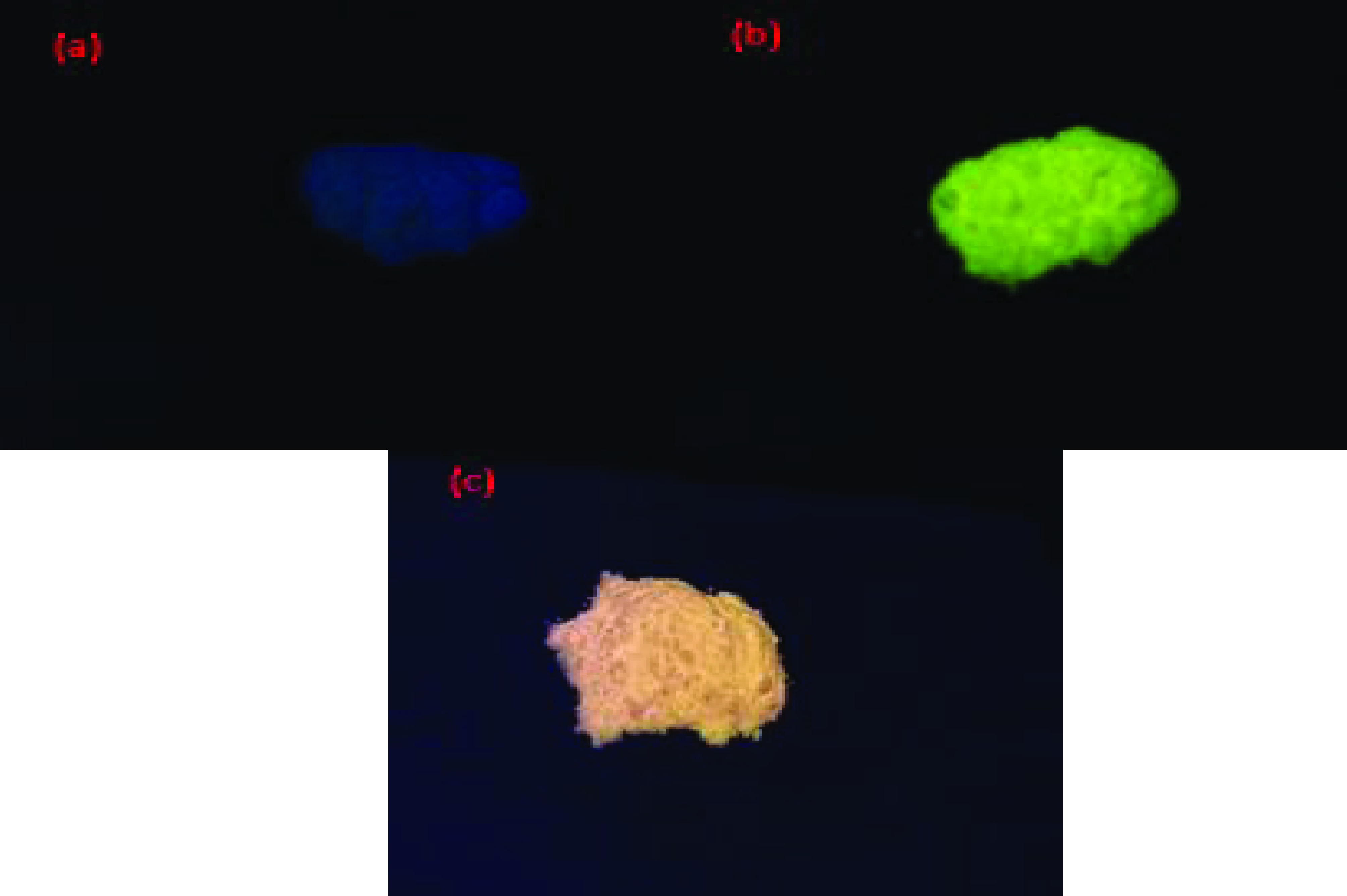
Photographs of nano-HAP (a), nano-HAP-F (b), and nano-HAP-Rd (c) under a UV lamp.

As shown in Figure 7, when the samples were excited under UV light (365 nm), it was clearly observed that nano-HAP had very low fluorescence (Figure 7a) when compared to those trapping fluorophores. The green (Figure 7b) and pink (Figure 7c) colors appeared only for nanoparticles trapping F and Rd, respectively.

**Figure 8:**
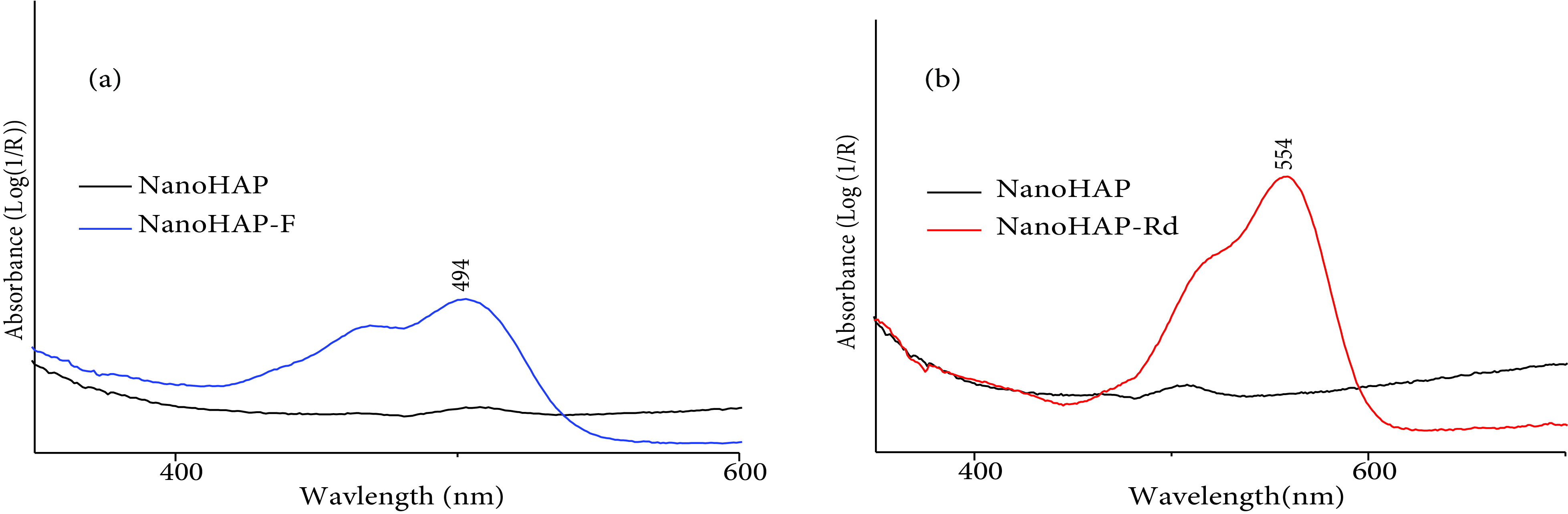
UV-Vis spectra of nano-HAP-F (a) and nano-HAP-Rd (b).

Nano-HAP, nano-HAP-F, and nano-HAP-Rd were analyzed via UV-Vis solid spectroscopy. The maximum absorption wavelengths were 494 nm and 554 nm, which corresponded to those of F and Rd, respectively. Figure 8 shows that the nano-HAP-F (Figure 8a) and nano-HAP-Rd (Figure 8b) spectra were more intense when compared to that of nano-HAP. The entrapment efficiency of F and Rd were about 65.17% and 90.76%, respectively. This efficiency was confirmed by UV-Vis spectroscopy by measuring the fluorophore content in the supernatant after centrifugation and filtration and then deducting the amount encapsulated by nano-HAP.

### 3.4. Study of fluorophore release (F and Rd)

To follow the kinetics of the release of F and Rd as a function of time, we first applied UV light. Next, the release of F and Rd was studied using UV-Vis spectroscopy.

#### 3.4.1. Observations under UV light

**Figure 9:**
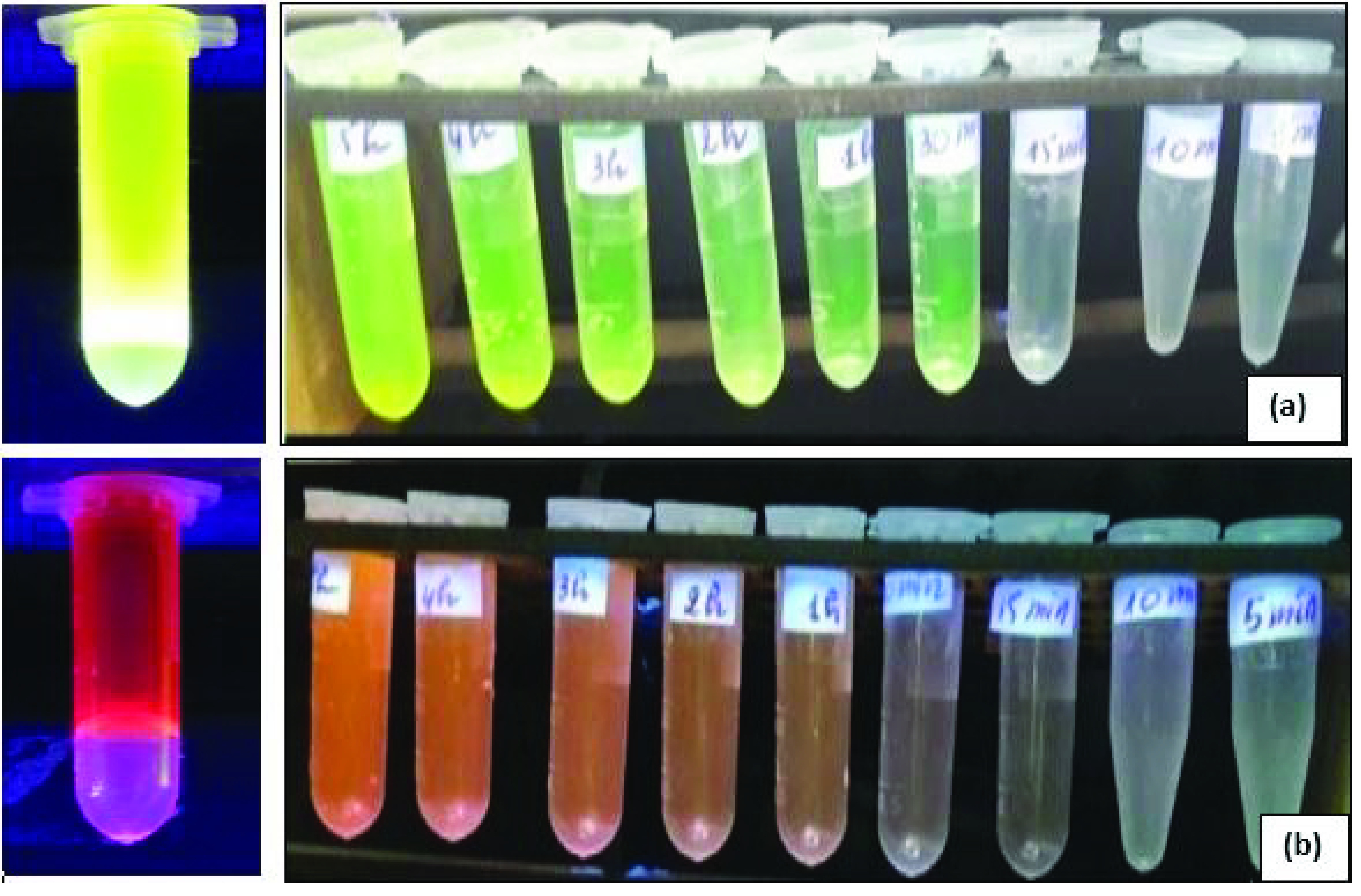
Observation of nano-HAP-F (a) and nano-HAP-Rd (b) under UV-light during the release of fluorophores as a function of time.

As illustrated in Figure 9, different colors were obtained by entrapping with different fluorophores. Samples were excited with UV light (365 nm). The calcium phosphate nanoparticles entrapping F emitted green light (Figure 9a) and the particles entrapping Rd emitted pink light (Figure 9b). The shade of the solution became darker over time, indicating that the fluorophore release prolonged over time. The prolonged release was confirmed by UV-Vis spectroscopy.

#### 3.4.2. UV-Vis analysis

The release of fluorophores was also studied via UV-Vis spectroscopy. Within a leaching time range of 5 min to 24 h for nano-HAP-F and 5 min to 4 days for nano-HAP-Rd, the maximum absorption wavelengths were 494 nm and 554 nm, respectively.

**Figure 10:**
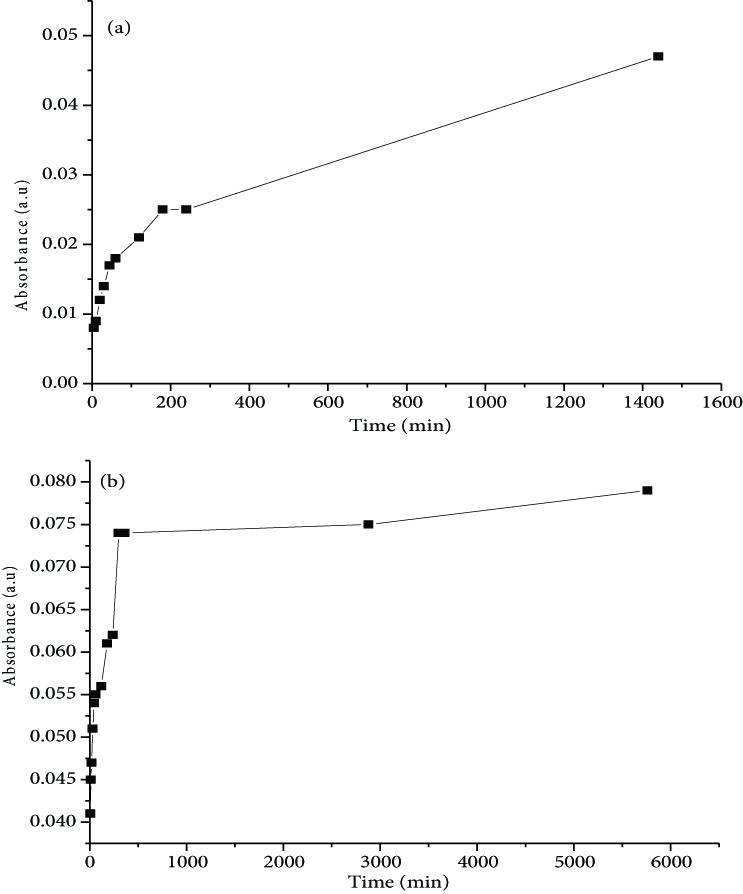
Release profile of (a) F and (b) Rd from nano-HAP-F and nano-HAP-Rd, respectively, over time, as measured by UV-Vis spectrophotometry.

The release efficiencies of F (Figure 10a) and Rd (Figure 10b) showed similar diffusional kinetic profiles, where the amount of released fluorophores increased as a function of time. As can be seen, the release of Rd was more sustained than that of F. This difference may have been due to the high solubility of Rd in water when compared to that of F.

### 3.5. Conclusions

To summarize, HAP nanoparticles were successfully prepared via the precipitation method. The average nano-HAP size evaluated from the TEM observations showed a proximate value of 20 nm. Nano-HAPs of such size and morphology are of interest for many applications for biological purposes, including optical imaging and photodynamic therapy. The prolonged release of fluorophores entrapped in nano-HAP will provide an interesting view of the roles of the crystalline phase of calcium phosphates for future clinical applications as ideal biomedical materials. Future work should focus on other ways to control the release of organic substances from these nanoparticles and the possibility of extending their release. In terms of size and trapping efficiency, and release of an external agent, these results were comparable to the findings of similar work, but considerably more effective than those synthesized by other groups [36–42].
